# Early Termination of a First-in-Human Trial of a Swallowable Self-Expanding Intragastric Balloon (IGBalloon©) Due to Small Bowel Obstruction

**DOI:** 10.1007/s11695-026-08845-5

**Published:** 2026-07-22

**Authors:** Daniel Moritz Felsenreich, Natalie Vock, Julia Jedamzik, Christoph Bichler, Magdalena Mairinger, Lisa Gensthaler, Larissa Nixdorf, Paula Richwien, Felix Benedikt Langer, Gerhard Prager

**Affiliations:** Division of Visceral Surgery, Department of General Surgery, Medical University of Vienna, Vienna, Austria

**Keywords:** Gastric balloon, Weight loss, IGBalloon©, Small bowel obstruction

## Abstract

**Background:**

Swallowable intragastric balloons represent an evolving minimally invasive treatment option for obesity. The current first-in-human study evaluated the safety and efficiency of a novel swallowable self-expanding intragastric balloon (IGBalloon©) with a kidney-shaped design, intended to reduce the risk of migration.

**Setting:**

Single-center, prospective first-in-human study at a tertiary metabolic/bariatric center.

**Methods:**

This prospective open-label, non-randomized trial was designed to include 20 patients with obesity. Patients with a body mass index (BMI) >35 kg/m² or >30 kg/m² with at least one obesity-related complication were eligible. The primary endpoints were safety and efficacy, including weight loss and remission of obesity-related complications. Patients were allowed to ingest up to five balloons during follow-up.

**Results:**

Due to two severe adverse events (SAE), the trial was terminated prematurely after the inclusion of five patients. Two patients developed small bowel obstruction caused by balloon migration through the pylorus, requiring emergency laparoscopic enterotomy and balloon removal.

**Conclusion:**

This first-in-human study demonstrated a substantial risk of balloon migration and small bowel obstruction associated with the current kidney-shaped IGBalloon© design. The device in its present form is not qualified for clinical use.

## Background

Obesity and its associated metabolic complications continue to represent a major global healthcare challenge. Current treatment strategies include lifestyle modifications, obesity management medications (OMMs), endoscopic bariatric therapies, and metabolic/bariatric surgery (MBS) [1–3]. MBS remains the most effective long-term treatment [4]. However, worldwide access remains limited, with only a small proportion of eligible patients ultimately undergoing surgery [5, 6].

Consequently, interest in less invasive obesity therapies has increased substantially during recent years [7].

In addition to pharmacotherapy [8], several endoscopic bariatric interventions have emerged as potential alternatives for selected patients [9, 10].

Intragastric balloons [11] have been used for several decades and have undergone multiple technical modifications regarding shape, filling mechanisms, implantation technique, and removal strategy [12]. Despite promising short-term weight-loss outcomes, a major limitation of most balloon systems remains weight regain following device removal [13].

Recently, swallowable balloon systems that do not require endoscopic placement have renewed interest in this therapeutic concept. The IGBalloon© was developed as a swallowable self-expanding balloon intended to remain within the stomach for prolonged periods without requiring complex implantation procedures. Its kidney-shaped design was specifically intended to reduce the risk of pyloric passage and distal migration.

Following successful preclinical testing, a prospective first-in-human trial was initiated to evaluate the safety and efficacy of this device. The present manuscript reports the study protocol, technical characteristics, clinical outcomes, and reasons for early trial termination following severe device-related complications.

## Methods

### Study Design

This study was designed as a prospective, non-randomized, open-label, first-in-human, single-center study. It was conducted to evaluate the safety and efficiency of the IGBalloon© in patients with obesity.

The study was planned to include 20 patients divided into two sequential cohorts:

- Group A (*n* = 5): intensive initial safety assessment.
- Group B (*n* = 15): subsequent efficacy evaluation.

Group B enrolment was planned to begin after completion of the initial 8-week safety assessment of Group A.

The primary study endpoints were:

1. Safety and device-related complications.
2. Weight-loss outcomes.
3. Remission of obesity-related complications.

Due to the occurrence of two severe adverse events requiring emergency surgery, the study was terminated prematurely after enrolling the first five patients.

### Patient Selection

Male and female patients aged 18–65 years with.

- BMI > 35 kg/m², or.
- BMI > 30 kg/m² with at least one obesity-related complication.

were considered eligible.

Obesity-related complications included:

- Type 2 diabetes mellitus.
- Arterial hypertension.
- Dyslipidemia.
- Obstructive sleep apnea.

Patients with previous metabolic/bariatric surgery were excluded. All participants provided written informed consent and agreed to participate in scheduled follow-up visits for at least one year.

### Study Timeline

The study was divided into a screening phase and a treatment phase. During the screening phase, patients swallowed an absorbable test balloon to assess swallowing capability and familiarize themselves with the procedure. In the treatment phase, patients ingested the activated IGBalloon©. Immediate post-placement gastroscopy was performed to confirm correct intragastric positioning and complete expansion.

Clinical follow-up visits were scheduled at.

- weeks 1, 2, 4, 6, and 8.
- Then at weeks 12, 16, 20, 24, 36, and 52.

**Fig. 1:**
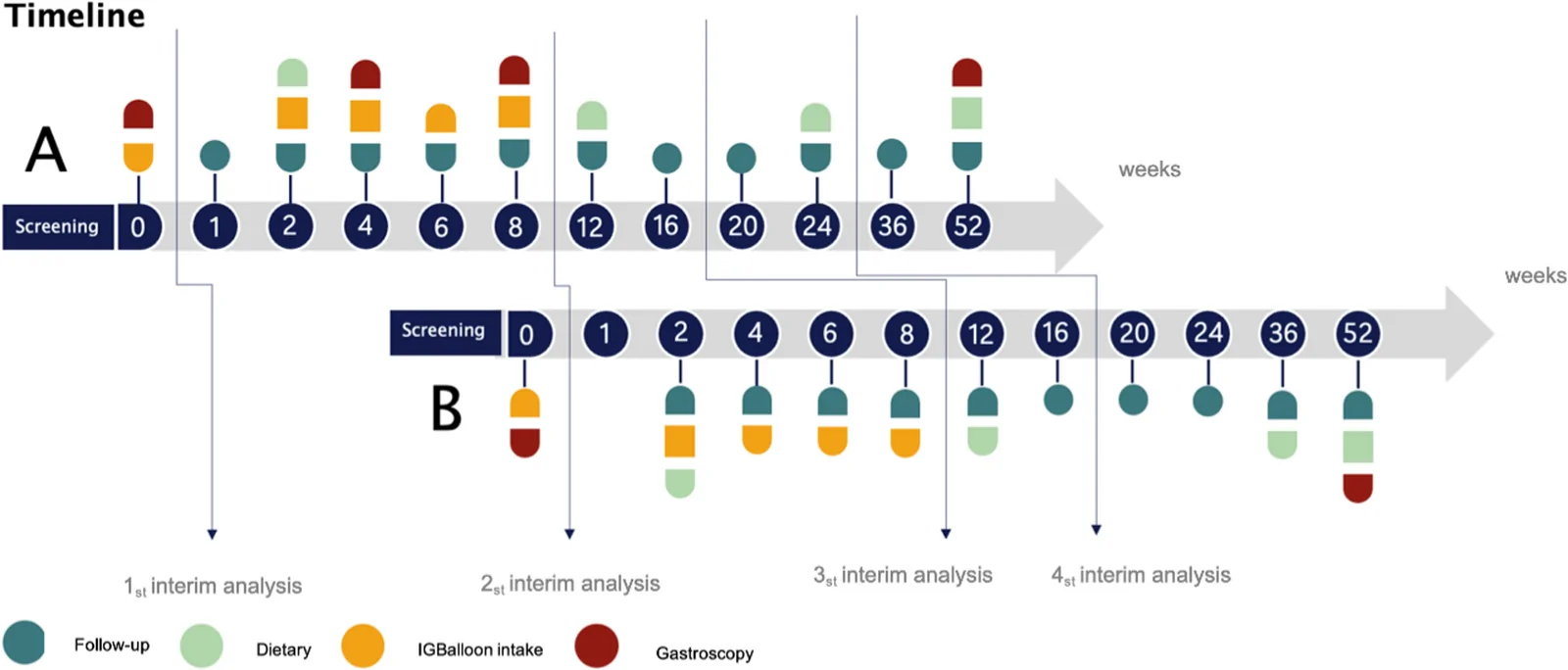
Planned timeline of the study

Patients were allowed to ingest additional balloons at weeks 2, 4, 6, and 8 based on satiety and weight-loss response, up to a maximum of five balloons. Participants had 24/7 access to the bariatric study team throughout the initial treatment period. A graphical depiction of the timeline can be seen in Fig. 1.

### Technical Characteristics of the Balloon

**Fig. 2:**
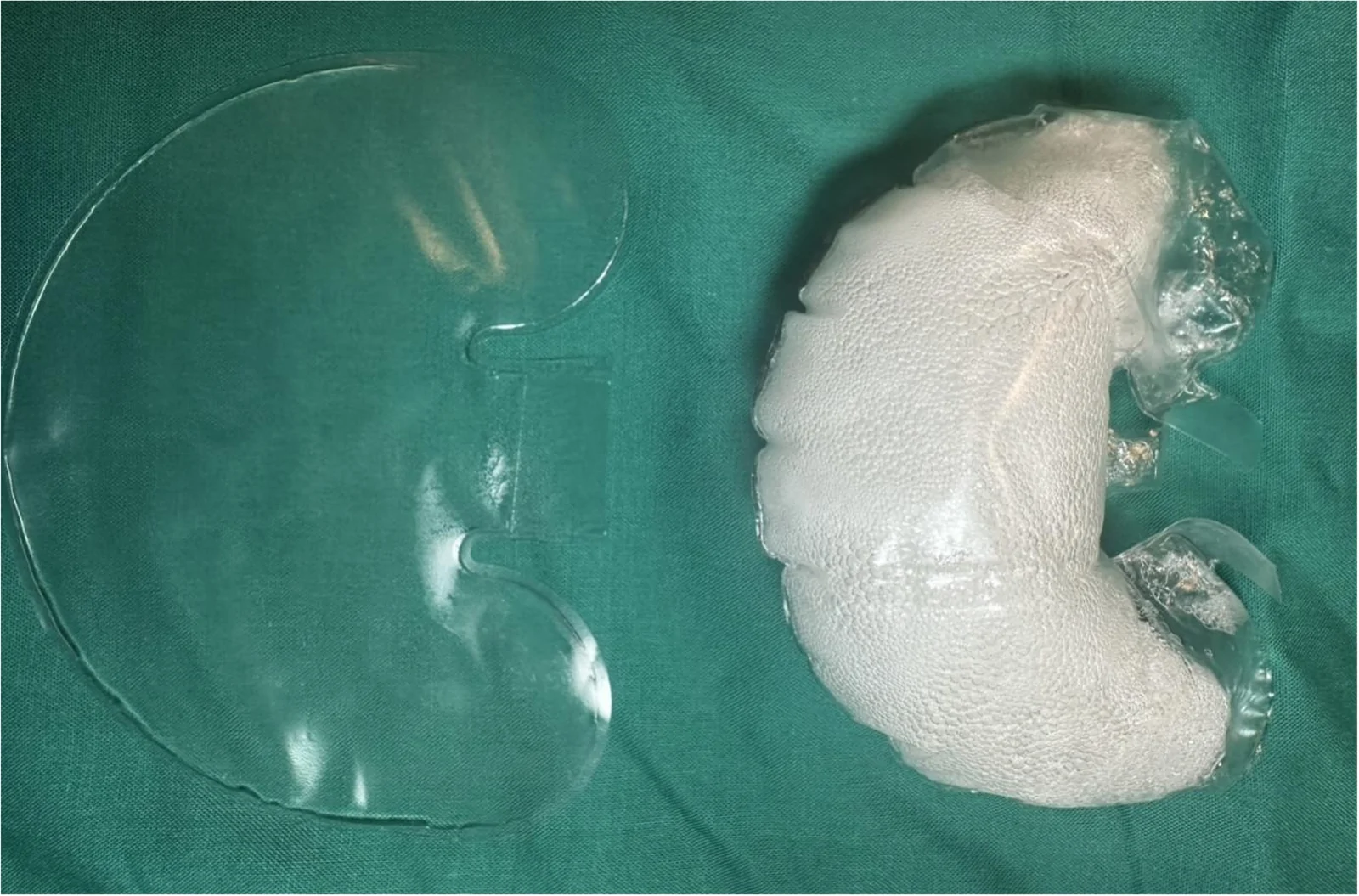
**a** Shape of the balloon before inflation; **b** Shape of the balloon in full extension

The IGBalloon© is a swallowable intragastric balloon consisting of an inert outer fluoropolymer membrane and internal medical-grade silicone (Fig. 2a and b). This device contains three separate silicone chambers, that are mixed immediately before ingestion initiating a temperature-dependent foaming reaction. Before activation, the balloon measures approximately 1 × 5 cm and contains approximately 12 ml of silicone fluid. Following ingestion and exposure to gastric temperature, the silicone expands into a three-dimensional kidney-shaped structure measuring approximately 10 × 7 × 5 cm with a final volume of approximately 120 ml.

The kidney-shaped design was intended to prevent migration through the pylorus and protect the gastric mucosa from potential filling port trauma. Furthermore, the solid elastomeric foam adheres to the inner membrane surface, theoretically reducing the risk of deflation after membrane damage.

### Endoscopic Evaluation

Gastroscopy was performed immediately after ingestion of the first IGBalloon© and during follow-up in accordance with the study protocol. Complete expansion and structural integrity of the balloon were assessed. Additional biopsies were obtained from the duodenum, antrum, corpus, and gastroesophageal junction. Histological evaluation included screening for helicobacter pylori. Any abnormalities detected during gastroscopy were documented, and further targeted biopsies were taken as indicated.

### Statistical Analysis

Because of early study termination after inclusion of only five patients, statistical analysis was limited to descriptive presentation. Continuous variables are reported as means ± standard deviations, whereas categorical variables are presented as percentages. No inferential statistical analyses were performed. Statistical analyses were performed using SPSS^®^ version 28 for Windows^®^ (IBM Corporation, Armonk, NY, USA).

## Results

### Baseline Characteristics

Of the planned 20 patients, 13 patients were screened and five patients initiated treatment before premature study termination.

The cohort consisted of four male and one female patients with a.

- mean age of 42.2 ± 11.4 years,
- mean weight of 121.8 ± 27.0 kg,
- mean BMI of 38.1 ± 5.3 kg/m²,

Baseline characteristics are summarized in Table 1. Mean follow-up duration was 56.8 ± 16.2 days.

**Table 1:** Baseline characteristics

	All patients (*n* = 5)
Sex (female) (*n* = 1) Sex (male) (*n* = 4) Age at procedure (years) Weight at procedure (kg) BMI at procedure ((kg/m^2^)	20% 80% 42.2 ± 11.4 121.8 ± 27.0 38.1 ± 5.3
Associated medical complications:	
Arterial hypertension Diabetes mellitus type 2 Obstructive sleep apnoea Cardiovascular disease GERD Disease of joints and bones Previous MBS procedures Nicotine abuses Mean follow-up (days)	40% 20% 20% 0% 60% 60% 0% 40% 56.8 ± 16.2

MBS: Metabolic/bariatric surgery; BMI: Body Mass Index; GERD: Gastroesophageal reflux disease

### Balloon Implantation and Follow-up

**Fig. 3:**
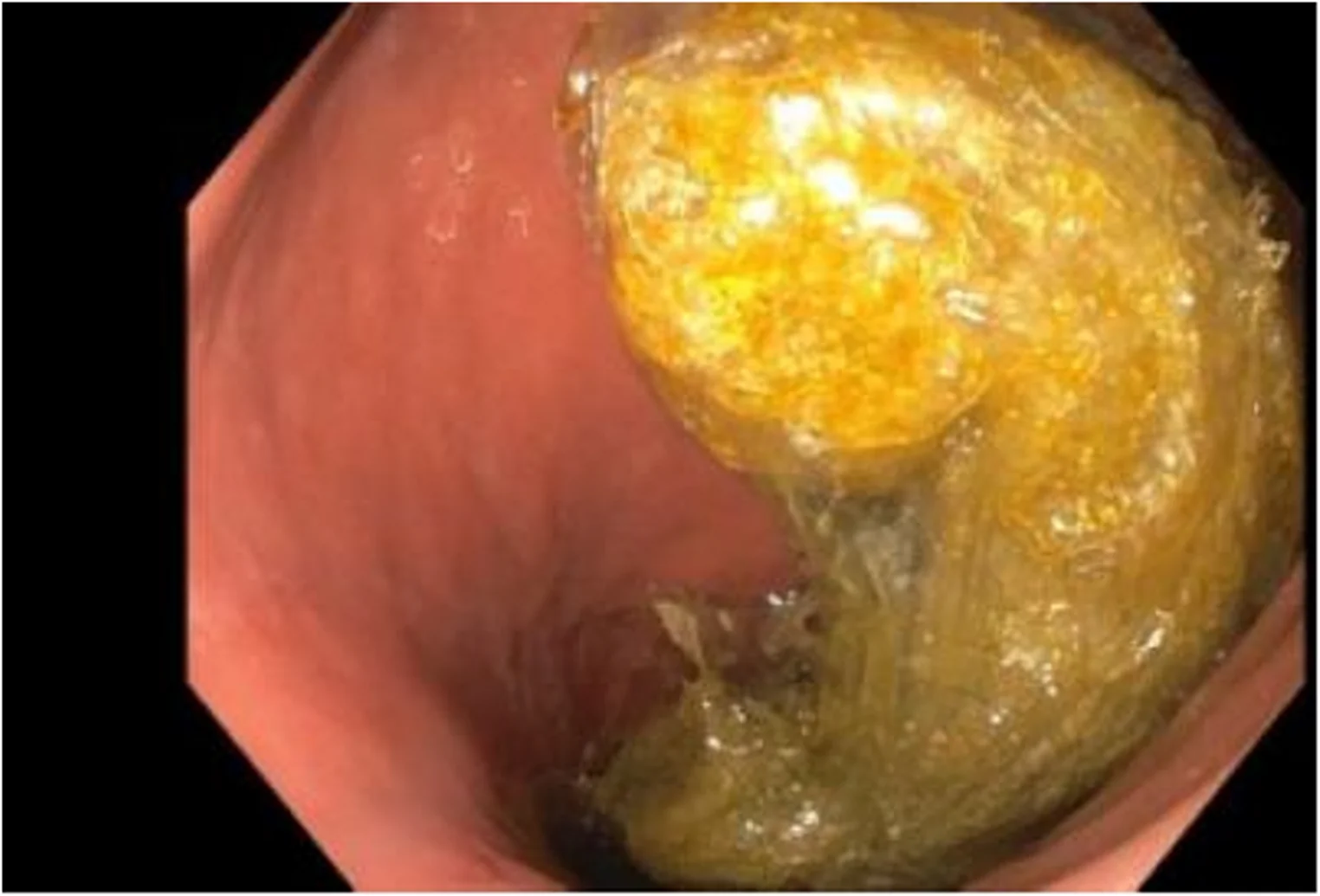
Balloon removal during emergency surgery

Two patients received one balloon, one patient received two balloons, and two patients received three balloons according to the study protocol. All patients successfully swallowed the activated balloons after prior training with the test balloons. No clinically relevant weight loss was observed during follow-up. For individual patient outcome see Table 2 (Fig. 3).

**Table 2:** Patient outcomes

Patient nr.	Weight week 0	BMI week 0	Follow-up (days)	Total number balloons	Weight follow-up	BMI follow-up	Side effects	Complications
1	127.9	41.3	61	3	129.6	42.3	Emesis Abdominal pain	Ileus*
2	133.0	36.8	58	3	133.0	36.8	Emesis Abdominal pain	Ileus*, wound infection**
3	159.6	46.6	68	1	160.0	46.7	non	none
4	110.7	35.3	68	1	110.0	35.1	GERD Diarrhea	none
5	83.0	32.0	29	2	82.0	31.6	Nausea	none

MBS: Metabolic/bariatric surgery; BMI: Body Mass Index; GERD: Gastroesophageal reflux

* Patients had emergency laparoscopy with enterotomy of the small bowel and balloon removal

** Wound infection with ambulatory treatment for five more weeks after discharge

### Adverse Events and Complications

**Fig. 4:**
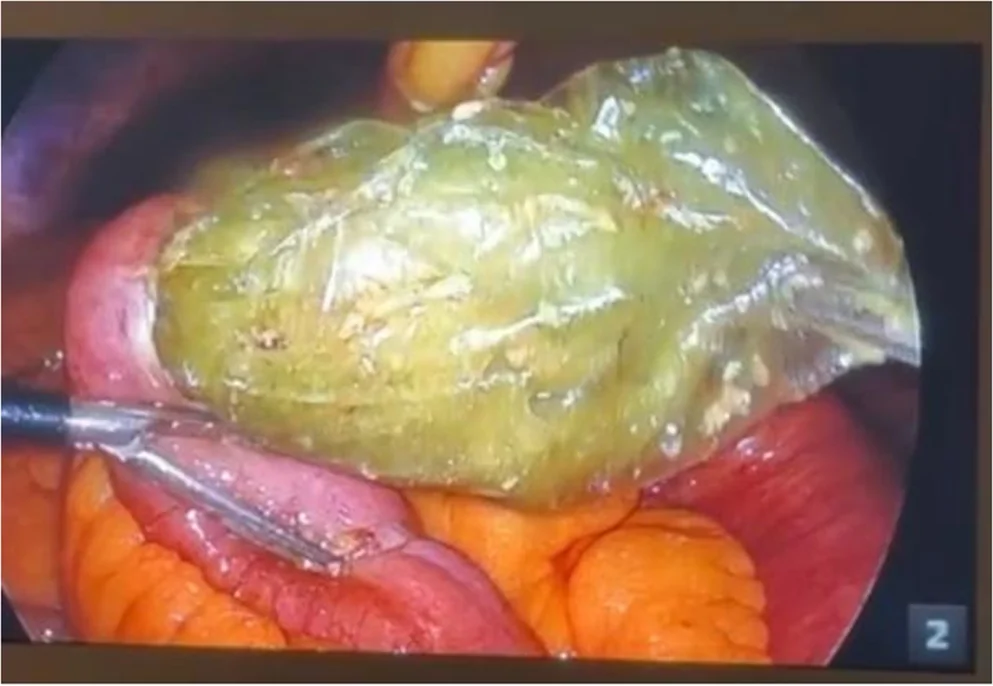
Bile in the balloon at the time of the removal

Minor adverse events included nausea, vomiting, abdominal pain, diarrhoea, and GERD. Two patients developed small bowel obstruction caused by balloon migration through the pylorus after 58 and 61 days, respectively. Both patients underwent emergency laparoscopy with enterotomy and balloon removal (Fig. 4). During the same procedure, the remaining intragastric balloons were removed endoscopically. One patient developed a severe wound infection requiring prolonged ambulatory treatment for five weeks following discharge.

Hospital stays were 8 and 13 days, respectively. Following the second severe adverse event, all remaining balloons in the other study patients were removed and the study was terminated immediately (Table 2).

### Endoscopic Findings

Initial gastroscopy demonstrated.

- mild gastritis in three patients (60%),
- grade-A esophagitis in three patients (60%),
- small hiatal hernia in one patient,
- helicobacter pylori infection in one patient.

During follow-up endoscopy, four of ten implanted balloons showed impaired membrane integrity with bile fluid entering the silicone matrix (Fig. 3).

## Discussion

This first-in-human study demonstrated a substantial safety concern associated with the current kidney-shaped IGBalloon© design. Two of five participating patients developed small bowel obstruction caused by balloon migration through the pylorus, requiring emergency surgical intervention. Consequently, the study was terminated prematurely. Although no conclusions regarding efficacy can be drawn because of the limited sample size and short follow-up, the absence of clinically relevant weight loss during the early treatment phase raises additional concerns regarding device performance.

### Device Migration and Balloon Design

The principal safety issue observed in this study was migration of the balloon through the pylorus despite its intended anti-migration kidney-shaped configuration. It is possible that the narrower component of the balloon aligned with the pyloric channel, thereby facilitating distal passage. Additionally, the elastomeric consistency of the silicone matrix may have allowed partial deformation during pyloric transit. These findings suggest that the current balloon geometry may be fundamentally unsuitable for reliable gastric retention.

Previous reports have described rare cases of migration and bowel obstruction even with conventional spherical balloons [14, 15]. However, the incidence observed in the present study was substantially higher, occurring in 40% of treated patients.

The inability to directly visualize the radiolucent balloon on computed tomography further complicated diagnosis. Future device iterations should therefore incorporate radiopaque markers to facilitate rapid identification in emergency settings.

### Swallowing and Removal Characteristics

Swallowing of the device proved feasible in all patients following prior test-balloon training. This represents an important advantage compared with endoscopically implanted balloon systems.

However, removal proved technically challenging. Because the balloon contained solidified silicone foam, volume reduction was not possible, necessitating extraction in full size using a large retrieval sling. Although no esophageal injury occurred, this technique may carry an increased theoretical risk of mucosal trauma. Compared with other swallowable systems featuring spontaneous deflation mechanism [16], the current device lacks a reliable non-interventional removal strategy.

### Comparison with Existing Balloon Systems

Several swallowable intragastric balloon systems have recently demonstrated promising short-term outcomes with acceptable safety profiles [16–18]. Meta-analyses report total weight loss ranging from approximately 12–16% after 4–10 months [17, 19]. In contrast, no meaningful weight reduction was observed in the present study. While interpretation is limited because of the short follow-up period, the absence of early weight loss despite multiple implanted balloons may indicate limited efficacy.

### Clinical Implications

The present study highlights the importance of rigorous safety monitoring during early-stage obesity device development. Transparent reporting of negative first-in-human experiences is essential to improve future device design and patient safety. Importantly, rapid access to a specialized bariatric center enabled prompt diagnosis and minimally invasive management in both cases. Delayed recognition in non-specialized settings could potentially have resulted in bowel ischemia, laparotomy, or increased morbidity.

### Future Perspectives

Despite the negative findings of the present trial, minimally invasive obesity therapies remain an area of major clinical interest. Future obesity treatment will likely involve multimodal strategies combining.

- lifestyle intervention,
- pharmacotherapy,
- endoscopic therapies,
- and metabolic surgery.

Swallowable endoscopic devices may still represent a promising therapeutic concept, if future generations achieve improved safety and efficacy profiles.

### Limitations

This study is limited by its very small sample size and early termination following severe adverse events. Consequently, no meaningful conclusions regarding efficacy can be drawn. Furthermore, as a first-in-human study, procedural learning curves and evolving patient selection may have influenced outcomes. Nevertheless, the severity and consistency of the observed complications strongly suggest a clinically relevant device-related safety issue.

## Conclusion

This first-in-human trial demonstrated a substantial risk of migration and small bowel obstruction associated with the current kidney-shaped IGBalloon© design. Two of five treated patients required emergency surgical intervention because of balloon migration through the pylorus. Although efficacy assessment was limited by early study termination, the present device configuration cannot be recommended for clinical use. The findings emphasize the importance of rigorous safety evaluation, transparent reporting, and careful device redesign during development of novel obesity therapies.

## Data Availability

All data supporting the findings of this study are available within the paper and its supplementary information.
